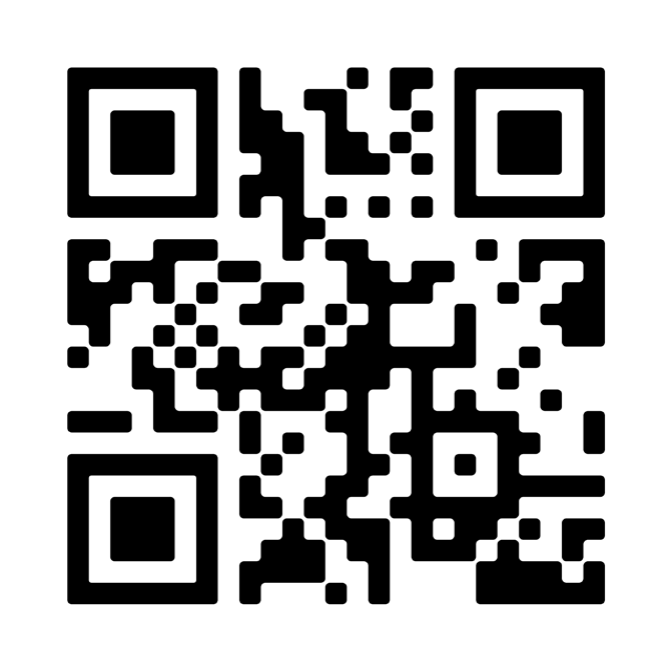# Indocyanine Green tattooing for marking the caudal excision margin of a full-thickness vaginal endometriotic nodule

**DOI:** 10.52054/FVVO.15.1.062

**Published:** 2023-03-31

**Authors:** S Khazali, B Mondelli, K Fleischer, M Adamczyk

**Affiliations:** HCA The Lister Hospital, Chelsea, London, United Kingdom; Royal Holloway – University of London, United Kingdom; Ashford and St Peter’s Hospitals NHS Foundation Trust, Chertsey, United Kingdom; Royal Surrey County Hospital, Guildford, United Kingdom

## Abstract

**Background:**

The use of Indocyanine Green (ICG) is well-described in oncology and more recently in benign gynaecological surgery. In this article we describe submucosal transvaginal ICG infiltration caudal to a vaginal endometriotic nodule to visualise the lower margin of excision laparoscopically.

**Objectives:**

To demonstrates the use of submucosal ICG tattooing to mark and delineate the caudal margin of an ultra-low full thickness vaginal nodule and aid its excision laparoscopically.

**Material and Methods:**

A stepwise approach highlighting the “SOSURE” surgical technique for the excision of endometriosis and the practical use of the ICG to delineate the lowest margin of the full thickness vaginal nodule.

**Main Outcome Measures:**

Laparoscopic complete excision of a 5 cm full-thickness vaginal nodule invading the right parametrium and involving the superficial muscularis layer of the rectum.

**Result:**

ICG tattooing was helpful in identifying the lower margin of dissection of the rectovaginal space.

**Conclusion:**

ICG tattooing of the margins of full-thickness vaginal nodules could be another use of ICG in benign gynaecology to complement the surgeon’s tactile and visual identification of the lower edge of dissection.

## Learning Objective

This video article aims to demonstrate the use of ICG to delineate the lower margins of a large 5 cm ultralow vaginal nodule extending its limits to 3 cm from the vaginal introitus. ICG tattooing facilitated identifying the caudal margin of excision.

## Introduction

Indocyanine green (ICG) has been widely used in gynaecological oncology to identify sentinel lymph nodes using near-infrared light (NIR light) (Lau and Estrada, 2020). In benign gynaecology, ICG can be used intravenously to assess the vascularisation of bowel anastomosis following segmental or disc resection (Blanco-Colino and Espin-Basany, 2018) and of ureters following ureterolysis. ICG can also be instilled into the ureteric lumen using a ureteric catheter to identify the course of the ureter laparoscopically (Kanammit et al., 2021). It can also be used instead of methylene blue (MB) in a number of scenarios (tubal patency test, bladder integrity test etc). More recently, the use of ICG in identification of uterine niches has been described (Krentel et al., 2022). The advantage of using ICG over methylene blue in these scenarios is the fact that ICG is transparent in normal light and does not stain the tissue. Submucosal ICG infiltration has been used for preoperative tattooing of rectal tumours (Lee et al., 2018).

Deep endometriosis involving the posterior compartment and invading the vagina constitutes a major challenge for the gynaecologist. When the vaginal nodule is very low, one of the recognised complications from excision is vaginal stricture and shortening. In order to minimise the dissection between the posterior vaginal wall and the rectum and reduce the margin of healthy vaginal tissue resected, ICG can be used to “tattoo” the tissue in order to identify lower border of the nodule.

## Patients and methods

The patient was a 26-year-old lady who attended our clinic with debilitating symptoms. These included premenstrual and menstrual pain, lower back ache and dyschezia.

Vaginal examination revealed a macroscopically visible 5 cm low full-thickness vaginal nodule, with the caudal border 3 cm from the introitus. The nodule was also invading into the superficial layers of rectal muscularis and was extending laterally involving the right parametrium and the posterior aspect of the cervix.

Preoperative evaluation included Magnetic Resonance Imaging (MRI) of the pelvis and assessment of the urinary tract. The case was discussed in the multidisciplinary team meeting and the patient was appropriately counselled following this. Ultimately the patient opted for surgical treatment, the risks of the procedure were carefully explained, including the significant risk of vaginal shortening, stricture, and cervical insufficiency.

The ‘SOSURE’ technique, previously described (Fleischer et al., 2021), was used to excise endometriosis. SOSURE is a mnemonic that provides a framework to describe a number of operative steps to maximise access and optimise assistance during endometriosis surgery. These steps include Survey and Sigmoid mobilisation, Ovarian mobilisation, Suspension of the uterus and the ovaries, Ureterolysis, Rectovaginal and pararectal spaces opening and ultimately Excision of endometriosis.

Before opening the rectovaginal space laparoscopically, we diluted 1 ampule of Indocyanine Green (which contains 25 mg of ICG) in 10 ml of Normal Saline and of this, 1 ml was injected trans-vaginally into the mucosa just below the nodule margin.

## Results

At laparoscopy, findings included superficial endometriosis overlying the pelvic sidewalls, mobile ovaries free of adhesions and a deep, 5 cm nodule involving the posterior/central compartments adherent to the rectum and obliterating the Pouch of Douglas. SOSURE steps were followed. Sigmoid attachments were divided. Ovaries were suspended using PDS 2-0 sutures and the uterus was suspended using a No. 1 Vicryl suture. Bilateral ureterolysis was performed and pararectal spaces were entered.

Just before opening the rectovaginal space, 1ml of diluted ICG was infiltrated into the vagina just caudal to the nodule. This allowed clear laparoscopic identification of the normal vaginal tissue caudal to the nodule while developing the rectovaginal space and facilitated precise excision without excessive dissection of healthy vaginal tissues.

The patient was discharged the day following surgery with oral analgesia and seven days of low molecular weight heparin prophylaxis. At six weeks review, the patient reported complete resolution of her symptoms.

## Discussion

Despite the lower margin being only 3cm from the vaginal introitus, a minimal amount of heathy vaginal tissue was excised during the treatment of this large, 5cm, rectovaginal nodule. The use of ICG complemented the surgeons’ intraoperative vaginal tactile assessment and facilitated the dissection and opening of the surgical planes.

In retrospect, a smaller volume than 1ml could have been injected for a more precise and sharper demarcation of the nodule. This would have allowed a reduced spillage of “green” and lighter stain of surrounding tissue around the nodule. With hindsight, approximately 0.1 – 0.2ml of ICG distributed in 3 or 4 different points along the lower margin of the vaginal nodule would have achieved a more accurate delineation of the disease.

For intraoperative purposes, NIR light (700- 900nm) is more advantageous than visible light due to its capability to penetrate deeper into tissue, up to 10 mm (van Manen et al., 2018).

Currently both ICG and MB are approved for clinical use by the Food and Drug Administration and the European Medicines Agency.

Indocyanine green is not only very safe (adverse events are reported in less than 1 in 40 000 patients and mostly comprise hypersensitivity reactions) (van Manen et al., 2018) but, also has the significant advantage over MB that it does not significantly stain the tissues. When the ICG mode is switched off on the imaging system, the green staining coming from the indocyanine green becomes scarcely visible, even when used at high concentrations. This opens a wide range of applications for indocyanine green when tattooing and marking tissue intraoperatively is required. We have used ICG in various scenarios, from assessing perfusion of the ureters following challenging ureterolysis and perfusion of a rectal anastomosis line, to ureter identification by ICG administration through a ureteric catheter, among other applications.

For the excision of the severe endometriosis, the importance of a structured approach in surgery cannot be overemphasised. It is important to note that the SOSURE technique is not designed to prescribe rigid pre-defined steps, but rather a framework the surgeon can use and modify accordingly to the particular surgical scenario (Fleischer et al., 2021).

## Conclusions

Indocyanine green can complement the surgeon’s tactile and visual feedback in the excision of these ultra-low vaginal nodules. In this case, tattooing the lower margin of an ultra-low full thickness vaginal nodule helped the recognition of the caudal margin of the vaginal nodule, hence avoiding unnecessary excision of normal vaginal tissue.

## Video scan (read QR)

https://vimeo.com/759465654/e159ddbb3d

**Figure:**